# Energy-Efficient Scheduling for Hybrid Tasks in Control Devices for the Internet of Things

**DOI:** 10.3390/s120811334

**Published:** 2012-08-17

**Authors:** Zhigang Gao, Yifan Wu, Guojun Dai, Haixia Xia

**Affiliations:** 1 College of Computer Science, Hangzhou Dianzi University, Hangzhou 310018, China; E-Mails: yfwu@hdu.edu.cn (Y.W.); daigj@hdu.edu.cn (G.D.); 2 College of Informatics & Electronics, Zhejiang Sci-Tech University, Hangzhou 310018, China; E-Mail: lansehaimj@163.com

**Keywords:** IoT, control devices, hybrid tasks, DVS, slowdown factors, slack time

## Abstract

In control devices for the Internet of Things (IoT), energy is one of the critical restriction factors. Dynamic voltage scaling (DVS) has been proved to be an effective method for reducing the energy consumption of processors. This paper proposes an energy-efficient scheduling algorithm for IoT control devices with hard real-time control tasks (HRCTs) and soft real-time tasks (SRTs). The main contribution of this paper includes two parts. First, it builds the Hybrid tasks with multi-subtasks of different function Weight (HoW) task model for IoT control devices. HoW describes the structure of HRCTs and SRTs, and their properties, e.g., deadlines, execution time, preemption properties, and energy-saving goals, *etc*. Second, it presents the Hybrid Tasks' Dynamic Voltage Scaling (HTDVS) algorithm. HTDVS first sets the slowdown factors of subtasks while meeting the different real-time requirements of HRCTs and SRTs, and then dynamically reclaims, reserves, and reuses the slack time of the subtasks to meet their ideal energy-saving goals. Experimental results show HTDVS can reduce energy consumption about 10%–80% while meeting the real-time requirements of HRCTs, HRCTs help to reduce the deadline miss ratio (DMR) of systems, and HTDVS has comparable performance with the greedy algorithm and is more favorable to keep the subtasks' ideal speeds.

## 1. Introduction

The term IoT first appeared in the white papers of Auto-ID Center about the Electronic Product Code (EPC) by Brock in 2001 [1]. From then on, IoT has been applied to areas far beyond logistics and permeated into every aspect of industrial production and our social lives, e.g., from industrial plants to healthcare, from assisted driving to intelligent homes [2]. Different from the traditional Internet which only connects computers, IoT implements widely interconnection between human beings, computers, and things, and provides intelligent computing and services for people [3].

Today, the IoT is regarded as an extension of ubiquitous computing [4]. Because of the need of improving ubiquitous ability or controlling effects, there are sensors, controllers and actuators in many IoT systems. In these IoT systems, sensing is often for the purpose of better controlling, and controlling is often for better sensing or working. For example, an Android mobile phone may have up to 10 kinds of sensors, such as accelerometers, gyroscopes, light sensors, *etc*. The Android mobile phone can control the status of its lenses, images, or menus to be convenient to use according to the values measured by its sensors. In fact, the above three components often consist of sensor/actuator networks (SANs). In SANs, the sensors and the actuators can communicate with the controllers by wired or wireless links. The sensors are often composed of nodes with low computing capability and low power. The sensors are used to monitor the states of the environment or physical systems, and send the sensed information to the controllers. Therefore, the sensors act as the bridges between physical and digital world [2]. The controllers are responsible for analyzing the information coming from the sensors, and recognizing the states of the environment or physical systems. After that, the controllers calculate the control parameters and send control commands to the actuators. The controllers usually have strong computing capability in order to run computation-intensive tasks. The actuators can adjust the states of the environment or physical systems by performing appropriate actions. In spite of being limited in number, the controllers play critical roles in SANs. On the one hand, they have important influence on the availability of SANs because they are the relay between the sensors and the actuators. On the other hand, they are closely involved in the control effects because multiple tasks may lead to larger response time jitters. In this paper, we call the controllers in SANs the control devices of the IoT. These devices are purely hard real-time systems from the point of view of traditional embedded systems. However, in the IoT, they are not isolated systems, but usually need to send information (e.g., states or feedback information) to and receive information (e.g., command information) from remote nodes or control centers, *i.e.*, they are hybrid systems with both HRCTs and SRTs. Besides inherent real-time constraints in control systems [5], because of the limitation of volume or weight, these devices usually have limited energy. Because the processors in controllers usually have powerful ability to carry out complex computing, the energy savings of processors is critical to lengthening the device lifetime and/or improving the flexibility of use. Hence, an energy-efficient scheduling method is necessary for the control devices of the IoT with energy constraints.

The existing energy-saving research usually aims at only hard real-time systems [6–14] or soft real-time systems [15–19], and does not consider improving the control performance of HRCTs during energy savings. Moreover, IoT control devices usually interact with external systems which have different speed requirements, while the existing research does not consider the diversity of subtasks' speed requirements. In this paper, we first present the HoW task model. In HoW, a task consists of multiple subtasks with different function weight and two kinds of energy-saving goals under the fixed priority scheduling mode. After that, we extend the method proposed in [20], and present a new energy-efficient uniprocessor scheduling method HTDVS for IoT control devices with HRCTs and SRTs. Under real-time constraints and different energy-saving goals, HTDVS can effectively save energy while reducing the response time jitters of HRCTs.

The work in this paper is suitable for IoT control devices for the following reasons:

- HoW simplifies task modeling and describes tasks' importance levels, energy characteristics and execution order. For example, besides algorithms, peripherals (such as integrated actuators and communication modules) can be modeled as interface tasks with spcific function weight and energy-saving goals. A process including information-acquiring, information-processing and command-sending can be modeled as a task with multiple subtasks.
- Energy can be saved by using HTDVS, which enhances the availability of SANs and improves the continuity of information from sensors.
- HTDVS reduces response time jitters, and improves the control quality for actuators.
- HTDVS guarantees the real-time requirements of tasks when there are both HRCTs and SRTs, which makes it meet the real-time requirements of control tasks.
- HTDVS tries its best to meet the speed requirements of subtasks, which makes it suitable for different speeds in communication links and peripherals.

The rest of this paper is organized as follows: Section 2 summarizes the related work. Section 3 presents the task model and processor model. The timing analysis technique is proposed in Section 4. In Section 5 we describe the implementation of energy-efficient speed control in design time and run time. Experiment results and analysis are given in Section 6. We conclude this paper in Section 7.

## 2. Related Work

This paper focuses on the energy-saving problem of IoT control devices, which is applied to ubiquitous sensing and controlling, and involves energy-saving and real-time requirements. In this section, we first introduce the research in ubiquitous sensing and controlling, and then review the energy-saving work in real-time systems.

### 2.1. Ubiquitous Sensing and Controlling

Sensor networks are the information sources of IoT control devices. Currently, many research efforts have been made on the energy-saving problems in Wireless Sensor Networks (WSNs). Chamam and Pierre presented a method to optimally plan the states of sensors in cluster-based sensor networks [21]. This method can generate an energy-optimal topology with longest network lifetime under the condition of full area coverage. Wang *et al.* studied the target tracking problem in WSNs [22], and developed an energy-efficient three-phase tracking approach in order to increase network lifetime. Xia *et al.* proposed the Energy-Efficient Opportunistic Localization (EEOL) method by waking up some a number of sensor nodes [23]. This method greatly reduces energy consumption while keeping high localization accuracy. In the existing research work of energy-saving problem in WSNs, a common assumption is there are a number of sensors with low computing capability and limited energy. The existing work usually focuses on controlling appropriate sensor nodes to be in active states and balancing the energy consumption in order to prolong the lifetime of the whole network, while not consider the real-time requirements of systems.

With the development of IoT, more and more research efforts are made on the ubiquitous sensing and controlling. Yoerger *et al.* reported an autonomous underwater vehicle called Autonomous Benthic Explorer (ABE) to carry out near-bottom surveys in deep sea [24]. ABE was equipped with a large number of sensors, such as the scanning and multibeam sonars, a magnetometer, a digital still camera, *etc.*, which made it suitable for conducting fine-scale quantitative surveys and mapping of the seafloor. They presented three algorithms in order to control the location of ABE and navigate during carrying out missions. Xia *et al.* presented a simple yet efficient method to deal with unpredictable packet loss on actuator nodes [5]. They used the PID algorithm to predict the control commands whenever data packets sensed are lost. This method guaranteed actuators produced control commands reliably and timely. Aroca *et al.* presented a simple but universal control architecture for low-cost mobile robots [25]. They used the sensors in both robots and computing devices (such as mobile phones and tablet computers) to obtain the states of robots or physical environment. The computing ability of computing devices is used to execute the control algorithms. The communication between a robot and a computing device was implemented through an audio interface. Their work mainly focused on the implementation of the audio interface between the robot and the computing device. Jing *et al.* presented a wearable gestural device named Magic Ring (MR) to implement the control of appliances [26]. MR served as a controller with sensing and controlling functions. MR had the ability to sense the gestures of a finger, recognize what control commands it represented, and send these control commands to the corresponding appliances. They emphasized on the architecture and gesture recognition of MR. Except for Xia *et al.* who considered the real-time requirement of communication [5], all of the above research efforts do not involve the real-time and energy-saving requirements.

In this paper, although we focus on the energy-saving problem of single processor with powerful performance by using DVS technique, the method presented in this paper can be easily integrated to a network with multiple controller and actuator nodes.

### 2.2. Energy Savings for Hard/Soft Real-Time Systems

Till now, there have been a large number of research efforts on applying DVS to real-time systems. The running voltages of tasks can be determined at design time (in a static manner) or run time (in a dynamic manner), or by combining the static and dynamic manners. On one hand, static DVS algorithms make use of tasks' information such as periods, deadlines, Worst-Case Execution Time (WCET), and other information to decide the supply voltages of tasks offline. On the other hand, dynamic DVS algorithms make use of runtime information of tasks to decide the supply voltages of tasks online.

In hard real-time systems, the timing requirements of tasks must be respected when energy is saved. Saewong and Rajkumar provided three static DVS algorithms [6]: the Sys-Clock algorithm, the PM-Clock algorithm, and the Opt-Clock algorithm. The Sys-Clock algorithm assigns a fixed processor voltage for a task set; the PM-Clock algorithm assigns a fixed voltage on the basis of every individual task; and the Opt-Clock algorithm tries to assign an optimal voltage for every task. Yao *et al.* proposed a static, optimal, and polynomial-time scheduling algorithm for aperiodic tasks [7]. Pillai and Shin first proposed a DVS algorithm based on schedulability tests [8], which selected a fixed supply voltage to meet all the deadlines of a task set under the Earliest Deadline First (EDF) or Rate Monotonic (RM) scheduling model, and then they presented two dynamic DVS algorithms. The first one is the Cycle-Conserving RT-DVS algorithm, which uses the information of the Actual Execution Time (AET) of the completed tasks under EDF scheduling, and the remaining time information of the running tasks under RM scheduling, to select the lowest feasible operating frequency. The second one is the Look-Ahead RT-DVS algorithm, which defers the execution of tasks as late as possible to make the energy consumption as little as possible. Zhu presented a novel approach combining feedback control with DVS schemes (Feedback-DVS) for hard real-time systems with dynamic workloads under EDF scheduling [9]. In Feedback-DVS, each task is divided into two portions. In the first portion, Feedback-DVS exploits frequency scaling when tasks execute with the average execution time in order to minimize the energy consumption of tasks. In the second portion, Feedback-DVS meets hard real-time requirements even in the worst execution time case. Chen and Thiele explored energy-efficient real-time task scheduling in leakage-aware DVS systems for homogeneous multiprocessor platforms [10]. They showed the critical speed might not be good enough to be viewed as the lowest speed of execution, and load balancing might not be good from the energy-saving point of view. Aiming at periodic tasksets, they developed a new algorithm (*i.e.*, the RSLTF algorithm) to perform processor and task assignment, which was more effective in energy savings. Although static DVS algorithms have few runtime overheads, there is almost no purely static DVS energy-saving algorithm for hard real-time systems. Static DVS algorithms take pessimistic views about the execution time of tasks in order to guarantee the hard real-time requirements of tasks, which will lead to pessimistic energy-saving results. In fact, the AET of tasks is usually less than the WCET of tasks [11,12], so there is more slack time to use in runtime to lower the voltage of a processor further. Most of the existing research uses dynamic DVS algorithms [9,10], or combines static DVS and dynamic DVS algorithms [6–8,13,14].

Energy-saving problem has also been widely researched in soft real-time systems where there are usually the requirements of DMR. Kargahi and Movaghar presented a method for reducing the long run power consumption of a soft real-time system using DVS [15]. They modeled the system by using a continuous-time Markov process model and gave a predefined upper bound on the fraction of lost jobs. Aiming at the voltage assignment with probability (VAP) of soft real-time embedded systems with uncertain execution times and discrete voltage DVS processors, Qiu *et al.* used Probabilistic Data-Flow Graph to model tasks and proposed two optimal algorithms [16], VAP_S for uniprocessors and VAP_M for multiprocessors to minimize the expected value of total energy consumption while meeting timing constraints with guaranteed confidence probabilities. Rusu *et al.* evaluated several DVS algorithms for systems with unpredictable workloads [17]. They found the stochastic DVS scheme based on the collected statistical information about the workload probability distribution was simple and effective to obtain the DVS scheduling and better explore power-performance tradeoffs. Kluge *et al.* presented the autocorrelation clustering (ACC) method to optimize the energy consumption of periodic soft real-time applications [18]. By learning the workload of single iterations within a higher period, ACC predicts future workloads to adjust clock frequency and scheduling parameters, which is completely application-independent. Yuan and Nahrstedt presented an energy-efficient scheduler named GRACE-OS for soft real-time multimedia applications to save energy [19]. GRACE-OS obtains demand distribution via online profiling and estimation, and then makes scheduling decisions based on the probability distribution of applications' cycle demands. It saves energy significantly, and guarantees the quality of service of applications by limiting their DMR. In the current research on DVS for soft real-time systems, static DVS algorithms are usually used for soft real-time systems when their runtime characteristics, such as the probabilities of execution time and/or the execution paths of tasks are known a priori, and dynamic DVS algorithms are usually used to set the voltages of tasks by predicting tasks' workload online. Similar to that in hard real-time systems, most of the existing research uses dynamic DVS algorithms [15,17–19].

In this paper, we present an energy-saving method for IoT control devices with hybrid tasks. Our research is different from the existing ones in the following points. First, it is required to meet both hard real-time requirements and soft real-time requirements, rather than merely one of them. Second, tasks have two energy-saving goals, rather than merely the lowest, the best as usual. Third, tasks have different function weight, and they are not so important as each other. Fourth, HRCTs have the requirements for reducing response time jitters.

## 3. System Model

In this section, we introduce the task model and processor model that will be used throughout this paper.

### 3.1. Task Model

In this subsection, we define the HoW task model. Under HoW, a system is composed of *n* periodic tasks, represented as *Γ* = {*τ_1_*,···, *τ_n_*}. A task *τ_i_* is represented as a five-tuple {*T_i_, D_i_, C_i_, C_B_, TY_i_*}, where *T_i_* is its period, *D_i_* is its relative deadline, *C_i_* is its WCET, *C_B_* is its best-case execution time (BCET), and *TY_i_* is its task type. There are two kinds of tasks in HoW according to their real-time requirements.

- HRCTs. If *τ_i_*_∈_ HRCTs, its worst-case response time (WCRT) *R_i_* must be no more than *D_i_* in order to meet its deadline requirement, and should be as close as possible to *D_i_* in order to improve control effect [27].
- SRTs. If *τ_i_*_∈_ SRTs, *R_i_* should try to be no more than *D_i_*, but this is not a mandatory requirement. *R_i_* can also be more than *D_i_* in order to improve CPU utilization and/or save more energy.

The set of all HRCTs is denoted as *Γ^H^*, and the set of all SRTs is denoted as *Γ^S^*. If *τ_i_* is a hard real-time control task, *TY_i_* is *true*. Otherwise, *TY_i_* is *false*.

A task *τ_i_* consists of *m* subtasks, *i.e., τ_(i,1)_*,···, *τ_(i,m)_*. Because of the interaction between processors and exterior components or exterior environment, the energy-saving behavior of tasks is not only restricted by the real-time requirements of tasks, but also influenced by other factors. For example, if a subtask *τ_(i,k)_* receives data from a sensor node, the execution speed of *τ_(i,k)_* is constrained by the speed of the data transmission of the sensor node. A suitable execution speed is favorable for the sensor node to avoid staying in the active state for a long time. Moreover, subtasks may have different importance levels when meeting their execution speeds. For example, a subtask which collects solar energy is more important than a subtask which shows its work state in a solar-powered device. In this paper, we use function weight to denote the importance level of a subtask when meeting their execution speeds.

The subtask *τ_(i,k)_* (1 ≤ k ≤ m) is characterized by a seven-tuple {*C_(i,k)_, C_B(i,k)_, P_(i,k)_, PP_(i,k)_, W_(i,k)_, EG_(i,k)_, S_idl(i,k)_*}, where *C_(i,k)_* is its WCET, *C_B(i,k)_* is its BCET, *P_(i,k)_* is its priority, *PP_(i,k)_* is its preemptive property, *W_(i,k)_* is its function weight, *EG_(i,k)_* is its energy-saving goal.

The energy-saving goals of subtasks are classified into two classes:

- Optimal speed first, *i.e.*, G1-type. For a subtask with the G1-type energy-saving goal, *EG_(i,k)_* is *1*, and *S_idl(i,k)_* is its corresponding slowdown factor when runs at its ideal running speed. It is favorable for a G1-type subtask to run at the ideal running speed, and a higher speed is also acceptable. But it is unnecessary to run at a speed below its ideal running speed.
- Lower speed better, *i.e.*, G2-type. For a task with the G2-type energy-saving goal, *EG_(i,k)_* is *2, S_idl(i,k)_* is the slowdown factor when the processor runs at its lowest running speed, and a higher speed above the lowest speed of the processor is also reasonable. Moreover, a lower speed is better.

If *τ_i_* is a HRCT, *PP_(i,m)_* is *false* (*i.e., τ_(i,m)_* is a non-preemptive task), and *C_(i,m)_* is equal to *C_B(i,m)_* (Note that we can make *C_(i,m)_* equal to *C_B(i,m)_* by properly partitioning tasks). For any other *τ_(i,k)_, PP_(i,k)_* is *true* (*i.e., τ_(i,k)_* is a preemptive task). One instance of *τ_i_* is generated every *T_i_* intervals, called a *job* of *τ_i_*. *τ_(i,k)_* has the same period as that of *τ_i_*, and the WCET/BCET of *τ_i_* is equal to the sum of the WCET/BCET of all *τ_i_*'s subtasks. After a job of *τ_i_* is released, the job's subtasks are executed sequentially, that is to say, the subtask *τ_(i,k + 1)_* will only be released after the subtask *τ_(i,k)_* is completed. Moreover, the *(q + 1)^st^* job of *τ_i_* will only be executed after the *q^th^* job of *τ_i_* completes. We assume no subtask can be included in more than one task; there is no blocking time caused by resource sharing among subtasks of different tasks; and system overheads, such as scheduler and context-switching overheads, are ignored.

Note that two points should be put forward. First, in IoT control devices, although the majority of tasks are periodic ones, there are some aperiodic tasks and sporadic tasks triggered by external physical events or commands. In our current research, we do not consider the aperiodic tasks and sporadic tasks. Although aperiodic tasks and sporadic tasks can be modeled as SRTs and HRCTs respectively, it will lead to pessimistic results in scheduling and energy savings because of the using of their minimal trigger intervals. In scheduling, combining bandwidth reservation with slack stealling may be a promising solution in our future research. Second, although we do not limit the DMR of SRTs in this paper, DMR can be controlled by monitoring the runtime DMR of SRTs and adjusting the slowdown factors of SRTs.

### 3.2. Processor Model

Currently, the vast majority of modern processors are made using Complementary Metal-Oxide-Semiconductor (CMOS) technology. The power consumption of a processor consists of two parts. One is the dynamic power consumption *P_D_* caused by the charging and discharging of gates on the circuits. *P_D_* can be modeled as a convex function of the processor speed, denoted as: $P_{D}(s)=C_{\text{eff}}V_{dd}^{2}s$ [28], where *s* = *k*(*V_dd_* − *V_t_*)^2^/*V_dd_* is the processor speed, and *C_eff_, V_dd_, V_t_*, and *k* denote the effective switching capacitance, the supply voltage, the threshold voltage, and the design-specific constant (*V_dd_* ≥ *V_t_* ≥ 0, *k* > 0, and *C_eff_* > 0) respectively. The other is the leakage power consumption *P_L_* mainly coming from leakage current. The leakage power can be modeled as a constant when it does not change with temperature, or a linear function of temperature. Therefore, *P_L_* can be denoted as *P_L_*(*T*) = *βT* + *γ*, where *T* is the temperature of the processor, *β* and *γ* are two constants. The total power of the processor is:

(1) $$P=P_{D}(s)+P_{L}(T)=C_{\text{eff}}V_{dd}^{2}s+\beta T+\gamma$$

In this paper, we use the periodic task model and assume the processor is never shutdown. We ignore the influence of leakage power, and simplify the CPU power consumption to be:

(2) $$P=P_{D}(s)=C_{\text{eff}}V_{dd}^{2}s$$

A wide range of processors can run at a sequence of discrete operating frequencies with corresponding supply voltages. Changing the supply voltage of a processor will lead to the corresponding changes of the processor's operating frequency. We assume a processor *Pr_i_* has the parameters of {(*f_1_*,*V_1_*),···, (*f_max_*,*V_max_*)}, where *f_j_* is the operating frequency, and *V_j_* is the supply voltage. In this paper, subtasks are fundamental entities to which DVS is applied. We are only interested in the relative power consumption of subtasks when they run at different supply voltages. Therefore, we normalize the power to the maximum power consumption. If *Pr_i_* is running at *V_i_*, its unit power consumption is defined as *V_i_^2^f_i_*/(*V_max_^2^f_max_*). Note that the WCET and the AET of a subtask *τ_(i,j)_* are all measured at the full speed of the processor where *τ_(i,j)_* resides.

## 4. Timing Analysis Technique

In this section, we first introduce the analysis method of tasks' schedulability and some basic notations used in the schedulability analysis, and then present the strategies for controlling the response time jitters of HRCTs and prove the upper bound of tasks' WCRT in HoW.

### 4.1. Timing Analysis Algorithm

We call the timing analysis algorithm presented by Harbour, Klein, and Lehoczky the HKL algorithm [29]. In [30], we extend the HKL algorithm and present a schedulability analysis method for a task set whose tasks have both preemptive subtasks and non-preemptive tasks. In this paper, we use the timing method in [30]. For the convenience of understanding, we introduce some basic notations which will be used in the timing analysis of this paper.

Let's assume *P_min(j)_* refer to the minimum priority of all the subtasks of *τ_j_*. When *P_min(j)_* > *P_(i,k_*_)_, *τ_j_* has *multiply preemptive effect* on *τ_(i,k)_*. If $\exists l,(P_{\left(j,1\right)},\cdots ,P_{\left(j,l\right)}>P_{\left(i,k\right)})\wedge (P_{\left(j,l+1\right)}<P_{\left(i,k\right)})$, *τ_j_* has *singly preemptive effect* on *τ_(i,k)_*. *MP_(i,k)_* denotes the task set that has multiply preemptive effect on *τ_(i,k)_*. *τ_j_* has *blocking effect* on *τ_(i,k)_* if $\exists l,r,(P_{\left(j,l\right)}<P_{\left(i,k\right)})\wedge ((P_{\left(j,l+1\right)},\cdots ,P_{\left(j,r\right)})>P_{\left(i,k\right)})\wedge (P_{\left(j,r+1\right)}<P_{\left(i,k\right)})$. *SP_(i,k)_* denotes the task set that has singly preemptive effect on *τ_(i,k)_*. If *τ_j_* has multiply preemptive effect, singly preemptive effect, or blocking effect on *τ_(i,k)_*, we say *τ_j_* is a multiply preemptive task, singly preemptive task, or blocking task of *τ_(i,k)_*. If the priorities of several continuous subtasks of *τ_j_* are higher than *P_(i,k_*_)_, they are called an *H* segment; If the priorities of several continuous subtasks of *τ_j_* are lower than *P_(i,k_*_)_, they are called an *L* segment.

The canonical form of a task *τ_i_* is a task *τ_i_*′ whose subtasks maintain the same order, but have the priority levels that do not decrease. Harbour *et al.* proved that the completion time of *τ_i_* was equal to that of *τ_i_*′. When we calculate the WCRT of *τ_i_*, we need to consider the influence from other tasks. For a given canonical form subtask *τ_(i,k)_*′, other tasks can be classified into five types as follows.

- Type-1 tasks, *i.e.*, (***H***) tasks. All subtasks' priorities of a type-1 task are higher than *P_(i,k)_*′. Type-1 tasks have multiply preemptive effect on *τ_(i,k)_*′;
- Type-2 tasks, *i.e.*, (**(*HL*)^+^**) tasks (+ denotes one or more times). In type-2 tasks, an *H* segment is followed by an *L* segment. Type-2 tasks usually have singly preemptive effect on *τ_(i,k)_*′;
- Type-3 tasks, *i.e.*, (**(*HL*)^+^*H***) tasks. In type-3 tasks, an *H* segment is followed by an *L* segment except that the final segment is an *H* segment. Type-3 tasks usually have singly preemptive effect on *τ_(i,k)_*′. One of type-2 or type-3 tasks may have blocking effect on *τ_(i,k)_*′;
- Type-4 tasks, *i.e.*, **(*LH*)^+^*L*^0^** tasks (0 denotes zero or one time). In type-4 tasks, an *L* segment is followed by an *H* segment except that there is one or no *L* segment as its final segment. Type-4 tasks only have blocking effect on *τ_(i,k)_*′;
- Type-5 tasks, *i.e.*, (***L***) tasks. All subtasks' priorities of a type-5 task are lower than *P_(i,k)_*′. Type-5 tasks have no influence on *τ_(i,k)_*′.

The WCRT of *τ_i_* can be calculated as follows. First, convert *τ_i_* into *τ_i_*′. Second, classify other tasks into five types and calculate the busy period of *τ_i_*′. Third, calculate the response time of each job of *τ_i_*′ in its busy period in ascending order of subtasks. The longest response time of all *τ_i_*′'s jobs is the WCRT of *τ_i_*. More details about the HKL algorithm and the timing analysis for hybrid tasks can refer to [29] and [30] respectively.

### 4.2. WCRT Upper Bound of Tasks

**Figure 1.**
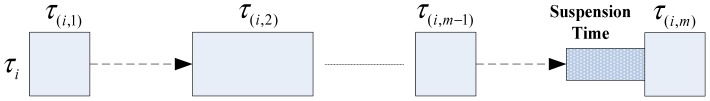
Structure of a HRCT *τ_i_*.

Many of the current embedded operating systems (OSs) (e.g., Windows CE, Linux, VxWorks, Android, iOS) provide self-suspension mechanisms to control the execution of tasks flexibly. In order to reduce the response time jitter of *τ_i_*, we insert a suspension time segment before *τ_(i,m)_*. The runtime structure of *τ_i_* is shown in Figure 1. In run time, the scheduler of an OS records the release time of a *τ_i_* instance, and inserts some a suspension time segment to make *τ_(i,m)_* suspend itself for a specific time. *τ_(i,m)_* can remove or effectively reduce its response time jitter because of the following reasons.

- The response time before the release of *τ_(i,m)_* can be measured by the scheduler;
- There is no task preemption between the end of the self-suspension and the end of *τ_(i,m)_* because the interrupt mechanism of OS and *τ_(i,m)_* is non-preemptive;
- Self-suspension time length can be set.

After using the self-suspension mechanism before the last subtasks of HRCTs, the response time of HRCTs and SRTs will change in comparison to that without the self-suspension mechanism. In this paper, we do not analyze the exact response time of a HRCT with a specific suspension time segment, and only give an upper bound of WCRT in order to guarantee the hard real-time requirements of HRCTs and try to meet the soft real-time requirements of SRTs.

In the following discussion, we call the task set where there is no suspension time segment *Γ^N^*, and *τ_i_^N^* denotes the task which has no self-suspension time segment. For example, if *τ_i_* belongs to *Γ^H^, τ_i_^N^* is the corresponding task of *τ_i_* which has no self-suspension segment in runtime; if *τ_i_* belongs to *Γ^S^, τ_i_^N^* is the same task as *τ_i_* whether at design time or run time. *I_i_^N^* denotes the interference time when the tasks having interference effect on *τ_i_* have their suspension time of zero. *IT_i_^N^* denotes the interference tasks of *τ_i_* in *Γ^N^*. *IT_i_* denotes the interference tasks of *τ_i_* in *Γ*. Let's prove the WCRT of *τ_i_^N^* in *Γ^N^* is the upper bound of WCRT of *τ_i_* in *Γ*.

#### Lemma 1

The interference time *I_i_* that *τ_i_* suffers from is no more than *I_i_^N^* when all of its interference tasks have no suspension time.

#### Proof

Without losing generality, we compare the interference effect from a HRCT *τ_j_*, and its corresponding task *τ_j_^N^*. From the task type classification, we know the following facts: (1) If *τ_j_* is a multiply/singly preemptive task of *τ_i_, τ_j_^N^* is still a multiply/singly preemptive task of *τ_i_*; (2) If *τ_j_* is a blocking task of *τ_i_, τ_j_^N^* is still a blocking task of *τ_i_*; (3) If *τ_j_* has no interference effect on *τ_i_, τ_j_^N^* has also no interference effect on *τ_i_*. So the tasks in *IT_i_* are the same as those in *IT_i_^N^*. If *τ_i_* is a HRCT, from [30,31], we know *τ_i_* has the same critical instant with tasks in *IT_i_* as with those in *IT_i_^N^*. Otherwise, it is obvious that *τ_i_* has the same critical instant with tasks in *IT_i_* as with those in *IT_i_^N^*. Because there is suspension time in *τ_j_*, the suspension time could be used to execute other interference tasks in *IT_i_* or *τ_i_*, which will make the length of *τ_i_*-busy period when *τ_i_* is executed with tasks in *IT_i_^N^* larger than or equal to that of *τ_i_*-busy period when *τ_i_* is executed with tasks in *IT_i_*. So the *τ_i_*-busy period when *τ_i_* is executed with tasks in *IT_i_^N^* is long enough to calculate the WCRT of *τ_i_*. Consider the scenario that the job of *τ_i_* has its WCRT, if the suspension time of some an interference task is filled with other interference tasks, the WCRT of *τ_i_* in *Γ* is equal to that of *τ_i_* in *Γ^N^*. Otherwise, the completion time of *τ_i_* will be sooner, *i.e.*, the WCRT of *τ_i_* in *Γ* will be less than that of *τ_i_* in *Γ^N^*. Therefore, the interference time *I_i_* that *τ_i_* suffers from is no more than *I_i_^N^* when all of its interference tasks have no suspension time, *i.e., τ_i_* will have the same or larger WCRT from *I_i_^N^* as or than that from *I_i_*.

#### Lemma 2

In *Γ^N^*, when converting a task *τ_i_^N^* into *τ_i_* by adding the suspension time segment of a HRCT, the WCRT of *τ_i_^N^* is no less than that of *τ_i_*.

#### Proof

Because the suspension time lies before the last subtask *τ_(i,m)_* of *τ_i_*, the multiply preemptive tasks, singly preemptive tasks and blocking task of *τ_i_* are the same as those of *τ_i_^N^. τ_i_* will also suffer from the maximum blocking time when it is released with its multiply preemptive tasks, singly preemptive tasks, and blocking task simultaneously, which is the same as that of *τ_i_^N^*. Because the suspension time segment of *τ_i_* will permit its interference tasks running during that time interval, the length of *τ_i_^N^*-busy period is no less than that of *τ_i_*-busy period. Because the suspension time of *τ_i_* is no more than *R_i_ − C_(i,m)_ − R_(i,m-1)_*, its suspension time can be filled with its interference tasks in the *τ_i_^N^*-busy period. Let's assume the WCRT of *τ_i_* is more than that of *τ_i_^N^*. If the job which has the WCRT is the first job in the *τ_i_*-busy period, it means the suspension time of *τ_i_* has been filled with its interference tasks. We may obtain larger or equal WCRT of *τ_i_^N^* when replacing *τ_i_* with *τ_i_^N^*, which contradicts the assumption. If the job which has the WCRT is not the first job in the *τ_i_*-busy period, it means the suspension time of the previous jobs of *τ_i_* has been filled with its interference tasks. Similarly, we can obtain larger or equal WCRT of *τ_i_^N^* when replacing *τ_i_* with *τ_i_^N^*, which contradicts the assumption, too. Therefore, In *Γ^N^*, when converting a task *τ_i_^N^* into *τ_i_* by adding some a suspension time segment, the WCRT of *τ_i_^N^* is no less than that of *τ_i_*.

From Lemma 1 and Lemma 2, we can easily derive theorem 1.

#### Theorem 1

Under the HoW model, the upper bound of WCRT of any task *τ_i_* in *Γ* can be derived from the corresponding task *τ_i_^N^* in *Γ^N^* while respects the deadline requirement of *τ_i_*.

Hence, we can use the method in [30] to analyze the schedulability of tasks in HoW.

## 5. Speed Assign Algorithms

**Figure 2.**
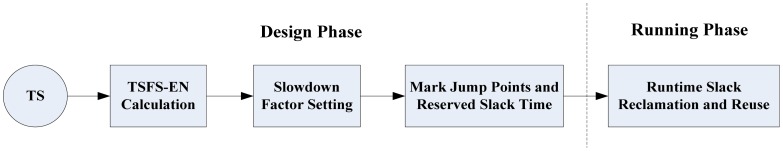
Process of assigning tasks' speeds.

Aiming at the energy-saving problem under the HoW task model, we present an energy-saving scheduling algorithm—the HTDVS algorithm. The HTDVS algorithm includes two phases, as shown in Figure 2. During design phase, it first calculates the Time Scaling Factors for a Single task/subtask Except Non-preemptive subtasks (TSFS-EN) factors of tasks (TSFS-EN Calculation) from a given task set TS. After that, it sets the slowdown factors of subtasks using hierarchical method (Slowdown Factor Setting). Finally, it marks the slack time restriction points of tasks and the reserved time requirements (Mark Jump Points and Reserved Slack Time). During the running phase, it reclaims and reuses the slack time of runtime subtasks according to the energy-saving goals of subtasks, dynamically sets the voltages of subtasks in order to save energy further, and sets the self-suspension time of HRCTs in order to reduce the response time jitters.

### 5.1. TSFS-EN and Slowdown Factors

In [20], Gao *et al.* presented the notation of Time Scaling Factors for a Single task/ subtask (TSFS). In this paper, we extend the time scaling factor to HoW, and present the notation of TSFS-EN. For a task *τ_i_* with a TSFS-EN of α, if we multiply every subtask's WCET of all tasks except their non-preemptive subtasks by a factor of α, *τ_i_* is still schedulable. The TSFS-EN factor of a task can be calculated using binary search.

From the definition of TSFS-EN, we know that the TSFS-EN of *τ_i_* only considers the schedulability of *τ_i_*, but not consider the influence of *τ_i_* on other tasks. Although there are two kinds of tasks, it only needs to restrict the interference time on HRCTs in order to guarantee the real-time requirements of HRCTs, and try its best to execute SRTs as slow as possible (of course, its speed is restricted by the slowdown factors and the energy-saving goals of its subtasks) in order to save more energy. In HoW, *τ_i_* may consist of more than one subtask. Because the interference on other tasks is based on that of *τ_i_'s* subtasks, we restrict the TSFS-EN of subtasks of *τ_i_* to avoid the pessimism of setting a minimum TSFS-EN for all subtasks of *τ_i_*.

In the following discussion, we denote the TSFS-EN of τ_i_ TSFS-EN(τ_i_). If τ_(i,k)_ has not been given an individual TSFS-EN, it uses the TSFS-EN of τ_i_ as its TSFS-EN. From the response time analysis of the HKL algorithm, we know all the multiply preemptive tasks and singly preemptive tasks of τ_j_ must fall into the set of MP_(j,1)_ (x0222A) SP_(j,1)_, that is to say, we can analyze the interference tasks of τ_j_ according to P_(j,1)_′. For the convenience of understanding, we introduce some notations in [20] which will be used in the following parts. When reducing τ_(i,k)_'s execution speed, we call the maximum allowable execution time of τ_(i,k)_ its maximum time extension. For an H/L task segment, its maximum time extension is equal to the sum of the maximum time extension of all its subtasks. The slowdown factor of τ_(i,k)_, denoted as S_(i,k)_, is defined as (τ_(i,k)_'s maximum time extension)/C_(i,k)_. Under the fixed priority scheduling and discrete voltage levels, assigning the optimal slowdown factors of tasks is an NP-hard problem [32]. In this paper, we do not make our effort to obtain the optimal slowdown factors of subtasks, but to assign the feasible and “effective” slowdown factors of subtasks. We assume T1(j) denotes the (**H**) task set of τ_(j,1)_′, T23(j) denotes the set of (**(HL)^+^**) and (**(HL)^+^H**) tasks of τ_(j,1)_′, and T234(j) is the set of (**(HL)^+^**), (**(HL)^+^H**), and (**(LH)^+^L^0^**) tasks of τ_(j,1)_′; $H_{i,j}^{I}$ denotes the initial H segment of τ_i_ against P_(j,1)_′; denotes the internal H segment to which τ_(i,k)_ belongs against P_(j,1)_′; $H_{i,j}^{L}$ denotes the final H segment of τ_i_ against P_(j,1)_′; $MTE_{(i,k),j}^{M}$ denotes the maximum time extension of $H_{(i,k),j}^{M};MTE_{i,j}^{L}$ denotes the maximum time extension of $H_{i,j}^{L}$. Let C_i_^h^ denote the WCET of the initial H segment of τ_i_; B_i_ denotes the maximum blocked time that τ_i_ can suffers from. For τ_i ∈_Γ, we set down the following three rules to restrict the interference time of τ_i_ on other task τ_j_ where *τ_j_* ∈ {Γ − *τ_i_* − Γ*^S^*}.≤

- Rule 1: if *τ_i_*_∈_*T1(j)*, for any subtask *τ_(i,k)_, S_(i,k)_* ≤ *TSFS-EN(τ_j_)*;
- Rule 2: if *τ_i_*_∈_*T23(j)*, for any *τ_(i,k)_*_∈_ $H_{i,j}^{I}$, S_(i,k)_ ≤ TSFS-EN(τ_j_);
- Rule 3: if *τ_i_*_∈_*T234(j), S_(i,k)_* ≤ *TSFS(τ_j_)*(C_i_^h^ + *B_j_*)/when *τ_(i,k)_*_∈_ $H_{(i,k),j}^{M}$;
  *S_(i,k)_* ≤ TSFS-EN(τ_j_)* $B_{j}/MTE_{i,j}^{L}$ when *τ_(i,k)_*_∈_ $H_{i,j}^{I}$ and *τ_i_*_∈_*Γ^S^*;
  *S_(i,k)_* ≤ (*B_i_* − *C_(i,m)_*)/(*MTEL i,j* − *C_(i,m)_*) (k < m) when *τ_(i,k)_*_∈_ $H_{i,j}^{L}$ and *τ_i_*_∈_*Γ^H^*.

**Figure 3.**
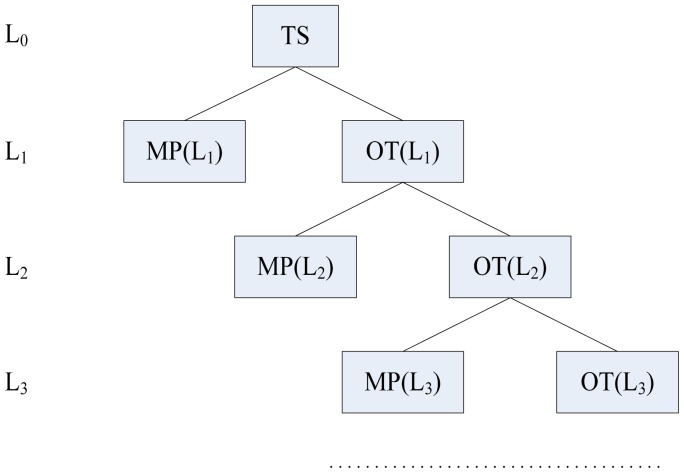
Task level tree in [20].

After that, we use the hierarchical tree method in [20] to classify tasks and set the slowdown factors of subtasks according to the above three rules. The classifying operation ends until there is no HRCT or only one task in TS or OT (other tasks except multiply preemptive tasks in a level). The structure of a hierarchical tree is shown in Figure 3. Note that the tasks with the minimum TSFS-EN in TS, MP and OT are only selected from *Γ^H^* because the real-time requirements of SRTs are not mandatory.

### 5.2. Setting Jump Points and Reserved Slack Time of Tasks

In [20], we have proven that the reclamation and reuse of slack time intra-a-task will not increase the WCRT of this task. However, it is necessary to restrict the maximum reusable slack time at jump points (*i.e.*, the first tasks of H segments after L segments).

Because real-time properties are not mandatory in SRTs, we only consider the jump points in HRCTs. From the classification of task types, we know the jump points of a task τ_j_ only appear in type-2, type-3, and type-4 tasks of τ_j_, *i.e.*, in T234(j).

If τ_(i,k)_ is a jump point of the internal H segment H_(i,k),j_^M^, the maximum reusable slack time (MRS) of τ_(i,k)_ is

(3) $${\text{MRS}}_{\left(i,k\right)}=\text{TSFS}-EN(\tau_{j})*(B_{j}+C_{i}^{h})-\sum_{\tau_{\left(i,p\right)}\in H_{(i,k),j}^{M}}S_{\left(i,p\right)}*C_{\left(i,p\right)}$$

If τ_(i,k)_ is a jump point of the final H segment H_i,j_^L^, the maximum reusable slack time of τ_(i,k)_ is

(4) $$MRS_{\left(i,k\right)}=\text{TSFS}-EN(\tau_{j})*(B_{j}+C_{\left(j,n\right)})+C_{\left(j,n\right)}-\sum_{\tau_{\left(i,p\right)}\in H_{i,j}^{L}}S_{\left(i,p\right)}*C_{\left(i,p\right)}$$

We adopt the method in [20] to set the jump points of tasks. Note that it only processes HRCTs. The slowdown factors and MRS of non-preemptive subtasks are set to 1 and 0 respectively.

The slack time requirements of τ_(i,k)_ are characterized by STIDL_(i,k)_, STMIN_(i,k)_, and STMAX_(i,k)_. STIDL_(i,k)_ denotes the ideal slack time that τ_(i,k)_ needs, STMIN_(i,k)_ denotes the minimum slack time that τ_(i,k)_ needs, and STMAX_(i,k)_ denotes the maximum slack time that τ_(i,k)_ needs. If τ_(i,k)_ is a G1-type subtask, STIDL_(i,k)_, STMIN_(i,k)_, and STMAX_(i,k)_ are all set to 0 if its ideal slowdown factor S_idl(i,k)_ is between 1 and S_(i,k)_. Otherwise, STIDL_(i,k)_ and STMAX_(i,k)_ are set to C_(i,k)_*(S_idl(i,k)_ − S_(i,k)_), and STMIN_(i,k)_ is set to C_B(i,k)_*(S_idl(i,k)_ − S_(i,k)_). If τ_(i,k)_ is a G2-type subtask, STIDL_(i,k)_ is set to 1/2*(C_B(i,k)_ + C_(i,k)_)*(S_min(i,k)_ − S_(i,k)_), STMIN_(i,k)_ is set to 0, and STMAX_(i,k)_ is set to C_(i,k)_*(S_min(i,k)_ − S_(i,k)_), where S_min(i,k)_ is the slowdown factor which is corresponding to the slowest speed of the processor.

In order to meet the real-time requirements and energy-saving requirements of tasks in HoW, it is necessary to mark the minimum reserved slack time (LRST), the ideal reserved slack time (IRST) and the maximum reserved slack time (MRST) of a subtask for its subsequent subtasks before using the runtime slack reclamation and reuse. For a subtask τ_(i,k)_, its LRST is:

(5) $$\text{LRS}T_{\left(i,k\right)}=\sum_{l>k\;\text{and}\;W_{\left(i,l\right)}>W_{\left(i,k\right)}}{\text{STMIN}}_{\left(i,l\right)}$$

where LRST_(i,k)_ is the LRST of τ_(i, k)_. Equation (5) means the LRST of τ_(i,k)_ is the sum of the STMIN of its subsequent subtasks with larger function weight. Similarly, IRST and MRST are defined as Equation (6) and Equation (7) respectively.

(6) $${\text{IRST}}_{\left(i,k\right)}=\sum_{l>k\;\text{and}\;W_{\left(i,l\right)}>W_{\left(i,k\right)}}{\text{STIDL}}_{\left(i,l\right)}$$

(7) $${\text{MRST}}_{\left(i,k\right)}=\sum_{l>k\;\text{and}\;W_{\left(i,l\right)}>W_{\left(i,k\right)}}{\text{STMAX}}_{\left(i,l\right)}$$

By scanning each subtask of all tasks, we can obtain their LRST, IRST, and MRST, and mark these information into the corresponding subtasks in order to reserve slack time for the following subtasks of a task.

### 5.3. Reclamation and Reuse of Slack Time in Runtime

In this section, we present two energy reclamation and reuse algorithms, *i.e.*, HTDVS-HRCT for HRCTs and HTDVS-SRT for SRTs respectively. By applying the two algorithms, the speeds of subtasks are adjusted dynamically according to their WCET, the H/L segments it belongs to, and the slack time it can use in run time. There are two kinds of slack time, *i.e.*, global slack time (GST) and local slack time (LST). GST of τ_i_, denoted as GST(i), is the slack time which is reclaimed in the executed subtasks of τ_i_ and can be used by all its subsequent subtasks. LST of τ_i_, denoted as LST(i), is the slack time which comes from GST(i) or is reclaimed in an H/L segment and can be reused in the same H/L segment.

The initialization, use and conversion rulers of LST and GST are as follows:

- Both LST and GST of a task are set to 0 before a task runs.
- LST are set in the first subtask of the first *H*/*L* segment, and all the slack reclaimed in a segment can be reused in the same segment.
- Before the first subtask of an *L* segment runs, all GST can be put into the LST of the first subtask. Before the first subtask of an *H* segment runs, the maximum value of LST can be equal to the maximum reusable slack time of the subtask, and it can extract slack from GST. Additional slack can be put into GST after an *H*/*L* segment completes.

On the basis of slack time reservation, the HTDVS-HRCT algorithm decides whether the subtask to be run can reuse the reclaimed slack time, and how much slack time the subtask can reuse. We define the following five rules when reusing slack time. Note that Rule 1 takes precedence over Rule 2.≥

- **Rule 1:** When *τ_(i, k)_* is a G1-type subtask, if *LST(i)* + *GST(i)* − *LRST(i,k)* ≥ *STIDL(i,k)* and *LST(i)* ≥ *STIDL(i, k*), *τ_(i, k)_* reuses the slack time of *STIDL(i, k)*, and runs at its ideal speed;
- **Rule 2:** For a G1-type subtask *τ_(i, k)_*, under the condition that *LST(i)* + *GST(i)* − *LRST(i,k)* ≥ *STMIN(i,k*) and *LST(i)* ≥ *STMIN(i,k), τ_(i, k)_* can reuse the slack time of *LST(i)* + *GST(i)* − *LRST(i,k)* and run at a lower speed if *LRST(i,k)* > *GST(i); τ_(i, k)_* can reuse the slack time of *LST(i)* and run at a lower speed if *GST(i)* ≥ *LRST(i,k)*;
- **Rule 3:** For a G2-type subtask *τ_(i, k)_* in an *H* segment, if *GST(i)* ≥ *IRST(i,k), τ_(i, k)_* can reuse the slack time of *LST(i)* and run at a lower speed; otherwise, if *LST(i)* + *GST(i)* ≥ *IRST(i,k)* and *IRST(i,k)* > *GST(i*), *τ_(i, k)_* can reuse the slack time of *LST(i)* + *GST(i)* − *IRST(i,k)* and run at a lower speed.
- **Rule 4:** For a G2-type subtask *τ_(i, k)_* in an *L* segment, if *GST(I)* ≥ *MRST(i,k), τ_(i, k)_* can reuse the slack time of *LST(i)* and run at a lower speed; otherwise, if *LST(i)* + *GST(i)* ≥ *MRST(i,k)* and *MRST(i,k)* > *GST(i), τ_(i, k)_* can reuse the slack time of *LST(i)* + *GST(i)* − *MRST(i,k)* and run at a lower speed.
- **Rule 5:** All subtasks which do not meet Rule 1–4 run at the speeds corresponding to their slowdown factors.

An HRCT can use the above five rules to reuse slack time. For a G1-type subtask τ_(i, k)_, using Rule 1, τ_(i, k)_ reserves LRST time for its subsequent subtasks and it runs at its ideal speed. Using Rule 2, τ_(i, k)_ reserves LRST time for its subsequent subtasks and it runs at a lower speed. By using Rule 1 and Rule 2, τ_(i, k)_ tries its best to work at its ideal speed or a lower speed above its ideal speed. For a G2-type subtask τ_(i, k)_, using Rule 3, τ_(i, k)_ in an H segment reserves IRST time for its subsequent subtasks and it runs at a lower speed. Because a subtask in an H segment is usually restricted in slack time reuse, it tries its best to reuse more slack time. Using Rule 4, τ_(i, k)_ in an L segment reserves MRST time for its subsequent subtasks, and τ_(i, k)_ runs at a lower speed. Because a subtask in an L segment is usually not restricted during slack time reuse, it tries its best to reserve more slack time. By using Rule 3 and Rule 4, τ_(i, k)_ will work at a lower speed after reserving enough slack time. Even if Rule 1–4 are not met, a subtask can reduce its running speed by using its slowdown factor. The HTDVS-HRCT algorithm is shown in Algorithm 1.

In Algorithm 1, Line 3–Line 47 are the pseudo code before releasing a subtask τ_(i, k)_, and Line 49 is the pseudo code upon τ_(i, k)_ ends. Line 3–Line 5 suspend the last subtask of τ_i_ for a specific time segment R_i_ − C_(i, m)_ − R_(i, m-1)_ according to the difference between R_i_ and R_(i, m − 1)_ in order to reduce the response time jitter of τ_i_. Line 7–Line 14 set the LST of τ_(i, k)_. If τ_(i, k)_ is in a jump point, *i.e.*, its restriction flag is true, its LST is set to its MRS, and GST are adjusted according to the slack time left. Otherwise, all slack time are assigned to the LST of τ_(i, k)_. Line 21 sets the runtime slowdown factor of τ_(i, k)_ to its static slowdown factor by default. If τ_(i, k)_ is a G1-type subtask, Rule 1 (Line 23–Line 24) or Rule 2 (Line 25–Line 29) can be used to calculate its runtime slowdown factor. If τ_(i, k)_ is a G2-type subtask, Rule 3 (Line 33–Line 37) or Rule 4 (Line 40–Line 43) can be used to calculate its runtime slowdown factor. The runtime voltage of τ_(i, k)_ is set in Line 46–Line 47. Upon task completion, LST is recalculated according to the running speed and AET of τ_(i, k)_ in Line 49.

On the basis of slack time reservation, the HTDVS-SRT algorithm decides whether the subtask to be run can reuse the reclaimed slack time, and how much slack time the subtask can reuse. We define the following four rules when reusing slack time.

- **Rule 1**: When *τ_(i, k)_* is a G1-type subtask, if *LST(i)* + *GST(i)* − *LRST(i,k)* ≥ *STIDL(i,k)*/2 and *LST(i)* ≥ *STIDL(i,k), τ_(i,k)_* reuses the slack time of *STIDL(i,k)*, and runs at its ideal speed;
- **Rule 2**: For a G1-type subtask *τ_(i, k)_*, under the condition that *STIDL(i,k)*/2 ≥ *LST(i)* + *GST(i)* − *LRST(i,k)* ≥ *STMIN(i,k)* and *LST(i)* ≥ *STMIN(i,k), τ_(i, k)_* can reuse the slack time of *LST(i)* + *GST(i)* − *LRST(i,k)* and run at a lower speed if *LRST(i,k)* > *GST(i); τ_(i, k)_* can reuse the slack time of LST(i) and run at a lower speed if *GST(i)* ≥ *LRST(i,k)*;
- **Rule 3**: For a G2-type subtask *τ_(i, k)_*, if *GST(i)* ≥ *IRST(i,k), τ_(i, k)_* can reuse the slack time of *LST(i)* and run at a lower speed; otherwise, if *LST(i)* + *GST(i)* ≥ *IRST(i,k)* and *IRST(i,k)* > *GST(i), τ_(i, k)_* can reuse the slack time of *LST(i)* + *GST(i)* − *IRST(i,k)* and run at a lower speed.
- **Rule 4:** All subtasks which do not meet Rule 1–3 run at the speeds corresponding to their slowdown factors.

**Algorithm 1.** HTDVS-HRCT algorithm.
**1****Algorithm HTDVS-HRCT**()/*τ_(i,k)_ is the subtask to be executed. *τ_(i,m)_* is the last subtask of *τ_i_*. *LST(i)* and *GST(i)* are the slack time before *τ_(i,k)_* is released. *f(V_(i,k)_′)* is the operating frequency corresponding to *V_(i,k)_′; AET_(i,k)_* is the actual execution time of *τ_(i,k)_*. *S_(i,k)_* is the slowdown factor of *τ_(i,k)_. S_(i,k)_′* is a temporary variable.*/2*Before task release:*3**if** ((*k* == *m*) and (*R_i_* − *C_(i,m)_* − *R_(i,m-1_*_)_ > 0)) {4  **Sleep**(*R_i_* − *C_(i,m)_* − *R_(i,m-1)_*);5  **return**;6 }7  **if (**the restriction flag of *τ_(i,k)_* is *true*) {8   **if** (*GST(i)* + *LST(i)* ≥ *MRS_(i,k)_*){9    *GST(i)* = *GST(i)* + *LST(i*) − *MRS_(i,k)_*;10    *LST(i)* = *MRS_(i,k)_*;11   }12  **else** {13   *LST(i)* = *LST(i)* + *GST(i)*;14   *GST(i)* = 0;15   }16  }17 **else** {18  *LST(i)* = *LST(i)*+*GST(i)*;19  *GST(i)* =0;20 }21 *S_(i,k)_′ = S_(i,k)_*;22  **if** (*τ_(i,k)_* is G1-type) {23   **if** (*LST(i)* + *GST(i) − LRST_(i,k)_* ≥ ***STIDL****_(i,k)_* and *LST(i)* ≥ ***STIDL****_(i,k)_*)24    *S_(i,k)_′*= *S_idl(i,k)_*;25   **else if** (*LST(i)* + *GST(i)-LRST_(i,k)_* ≥ ***STMIN****_(i,k)_* and *LST(i)* ≥ ***STMIN****_(i,k)_*) {26    **if** (*LRST_(i,k)_ > GST(i)*)27     *S_(i,k)_′* = (S_(i,k)_*C_(i,k)_ + *LST(i)* + *GST(i) − LRST_(i,k)_*)/*C_(i,k)_*;28    **else if** (*GST(i)* ≥ *LRST_(i,k)_*)29     *S_(i,k)_′* = (S_(i,k)_*C_(i,k)_ + *LST(i)*)/*C_(i,k)_*;30   }31  }32  **else if** (*τ_(i,k)_* is G2-type) {33   **if** (the *H* tag of *τ_(i,k)_* is true) {34    **if** (*LST(i)* + *GST(i)* ≥ *IRST_(i,k)_* and *GST(i) < IRST_(i,k)_*)35     *S_(i,k)_′*=(S_(i,k)_*C_(i,k)_ + *LST(i)* + *GST(i) − IRST _(i,k)_*)/*C_(i,k)_*;36    **else if**
*(GST(i)* ≥ *IRST_(i,k)_)*37     *S_(i,k)_′* = (S_(i,k)_*C_(i,k)_ + *LST(i)*)/*C_(i,k)_*;38   }39  **else** {40   **if** (*LST(i)* + *GST(i)* ≥ *MRST_(i,k)_* and *GST(i) < MRST_(i,k)_*)41    *S_(i,k)_′* = (S_(i,k)_*C_(i,k)_ + *LST(i)* + *GST(i) − MRST_(i,k)_*)/*C_(i,k)_*;42   **else** (*GST(i) ≥ MRST_(i,k)_*)43   *S_(i,k)_′* = (S_(i,k)_*C_(i,k)_ + *LST(i)*)/*C_(i,k)_*;44   }45  }46 **if** (*V_i_* is the least voltage that makes *f_i_*/*f_max_* equal to or larger than 1/*S_(i,k)_′*);47  Set the operating voltage *V_(i,k)_′* to *V_i_*;48*Upon task completion:*49 *LST(i)* = *LST(i)* + S_(i,k)_*C_(i,k)_ − *f_max_*/f(V_(i,k)_′)*AET_(i,k)_;


In Rule 1 and Rule 2, the purpose of setting a lower threshold for reserved slack time is to use more slack time to lower the running speed of a G1-type SRT's subtask. By using Rule 1 and Rule 2, a G1-type SRT's subtask τ_(i, k)_ will try its best to work at its ideal speed or a lower speed. Using Rule 3, a G2-type SRT's subtask τ_(i, k)_ reserves IRST time for its subsequent subtasks, and τ_(i, k)_ runs at a lower speed.

The HTDVS-SRT algorithm is shown in Algorithm 2. In Algorithm 2, Line 3–Line 35 are the pseudo code before releasing τ_(i, k)_, and Line 37 is the pseudo code upon τ_(i, k)_ ends. Line 3–Line 16 set the LST of τ_(i, k)_. Line 17 sets the runtime slowdown factor of τ_(i, k)_ to its static slowdown factors by default. If τ_(i, k)_ is a G1-type subtask, Rule 1 (Line 19–Line 20) or Rule 2 (Line 21–Line 25) can be used to calculate its runtime slowdown factor. If τ_(i, k)_ is a G2-type subtask, Rule 3 (Line 29–Line 32) can be used to calculate its runtime slowdown factor. The runtime voltage of τ_(i, k)_ is set in Line 34–Line 35. Upon task completion, LST is recalculated according to the running speed and AET of τ_(i, k)_ in Line 37.


**Algorithm 2.** HTDVS-SRT algorithm.
1**Algorithm HTDVS-SRT**()/*τ_(i,k)_ is the subtask to be executed. *τ_(i,m)_* is the last subtask of *τ_i_*. *LST(i) and GST(i)* are the slack time before *τ_(i,k)_* is released. *f(V_(i,k)_′)* is the operating frequency corresponding to *V_(i,k)_′; AET_(i,k)_* is the actual execution time of *τ_(i,k)_*. *S_(i,k)_* is the slowdown factor of *τ_(i,k)_. S_(i,k)_′* is a temporary variable.*/*2**Before task release:*3 **if (**the restriction flag of *τ_(i,k)_* is *true*) {4  **if** (*GST(i)* + *LST(i)* ≥ *MRS_(i,k)_*){5   *GST(i)* = *GST(i)* + *LST(i)* − *MRS_(i,k)_*;6   *LST(i)* = *MRS_(i,k)_*;7  }8  **else** {9   *LST(i)* = *LST(i)* + *GST(i)*;10   *GST(i)* = 0;11  }12 }13 **else** {14   *LST(i)* = *LST(i)* + *GST(i)*;15   *GST(i)* = 0;16 }17 *S_(i,k)_′= S_(i,k)_*;18 **if** (*τ_(i,k)_* is G1-type) {19  **if** (*LST(i)* + *GST(i) − LRST_(i,k)_ ≥*
***STIDL****_(i,k)_***/2** and *LST(i) ≥*
***STIDL****_(i,k)_*)20   *S_(i,k)_′* = *S_idl(i,k)_*;21  **else if** (*LST(i)* + *GST(i) − LRST_(i,k)_ ≥*
***STMIN****_(i,k)_* and *LST(i) ≥*
***STMIN****_(i,k)_*) {22   **if** (*LRST_(i,k)_ > GST(i)*)23    *S_(i,k)_′*=(S_(i,k)_*C_(i,k)_ + *LST(i)* + *GST(i) − LRST_(i,k)_*)/*C_(i,k)_*;24   **else if** (*GST(i) ≥ LRST_(i,k)_*)25    *S_(i,k)_′* = (S_(i,k)_*C_(i,k)_ + *LST(i)*)/*C_(i,k)_*;26  }27 }28 **else if** (*τ_(i,k)_* is G2-type) {29  **if** (*LST(i)* + *GST(i) ≥ IRST_(i,k)_* and *GST(i) < IRST_(i,k)_*)30   *S_(i,k)_′*=(S_(i,k)_*C_(i,k)_ + *LST(i)* + *GST(i)* − *IRST _(i,k)_*)/*C_(i,k)_*;31  **else if** (*GST(i) ≥ IRST_(i,k)_*)*32*   *S_(i,k)_′*=(S_(i,k)_*C_(i,k)_ + *LST(i)*)/*C_(i,k)_*;33 }34 **if** (*V_i_* is the least voltage that makes *f_i_*/*f_max_* equal to or larger than 1/*S_(i,k)_′*);35  Set the operating voltage *V_(i,k)_′* to *V_i_*;36 *Upon task completion:**37*  *LST(τ_i_)* = *LST(τ_i_)* + S_(i,k)_*C_(i,k)_ − *f_max_*/f(V_(i,k)_′)*AET_(i,k)_;


## 6. Experiments and Analysis

We developed a simulator using Visual C++ to test the HTDVS algorithm presented in this paper. This simulator can accept the speed/energy consumption function of a processor in its processor model and tasks' parameters in its task model as input, and analyze the energy consumption when using the HTDVS algorithm. In the following experiments, we use the method presented in [8] to generate the experimental task sets. The periods of tasks can be the short (1–10 ms), medium (10–100 ms), or long (100–1,000 ms) periods in order to simulate different kinds of applications. Tasks are generated randomly and uniformly distributed in the three kinds of periods. The WCET and BCET of the subtasks of a task *τ_i_* is generated randomly under the condition that *C_(i,k)_* is no less than *C_B(i,k)_*. When testing the energy consumption of a task set under a specific utilization, we first generate a task set with the maximum utilization 0.9, and then multiply the WCET and BCET of all tasks and its subtasks by a factor to obtain the task set under the specified utilization. After that, we use the uniform distribution function to generate the AET of subtasks. The AET of a subtask is uniformly distributed between its BCET and its WCET. In order to reduce error, we use the average value of ten experiments as the measure value of every data point. The total energy consumption of a task set is measured in [0, LCM], where LCM is the least common multiple of all tasks' periods.

Because the task model and scheduling model in this paper are different from those of the existing works in energy-saving research, we are not able to compare our method to other known DVS algorithms, such as the RT-DVS algorithm [8], the DRA algorithm [33], and the DVSST algorithm [34]. We performed three kinds of simulation experiments under the condition of different task type ratio, different energy reuse algorithms, and different subtask type ratio. In the following three experiments, we use the normalized speed/energy consumption parameters of Transmeta TM5800 processor {(1, 1), (0.9, 0.835), (0.8, 0.632), (0.667, 0.443), (0.533, 0.292), (0.433, 0.203), (0.3, 0.105)} as the processor parameters to calculate the energy consumption of tasks because it has fine and uniform speed-scaling granularity and can better reveal the energy characteristic of task sets [35]. We use the task sets generated randomly with the following parameters: number of tasks: 6; number of subtasks in each task: 2–5. When generating the task sets, all tasks are schedulable under the utilization of 0.9 and CPU speed of 1.

### 6.1. Different Task Type Ratio

**Figure 4.**
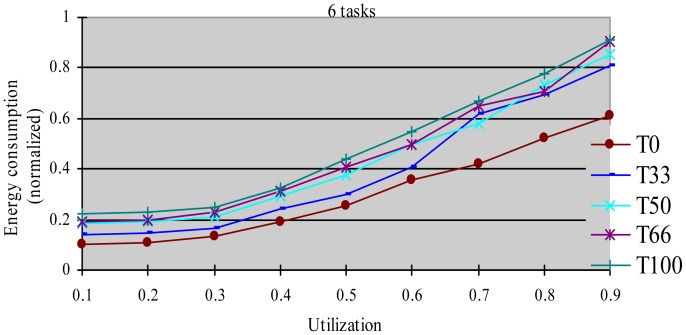
Normalized energy consumption at different task type ratio.

**Figure 5.**
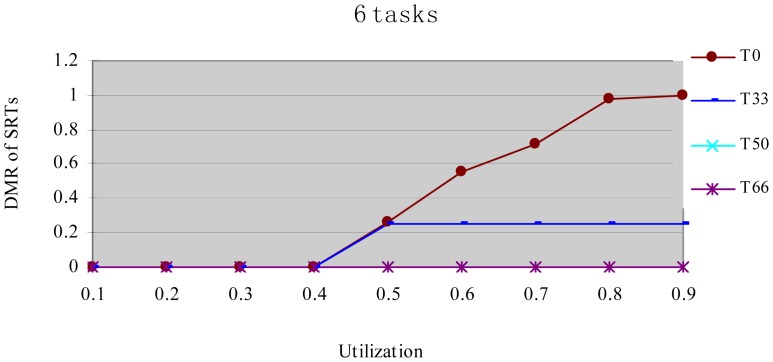
DMR of SRTs at different task type ratio.

In this experiment, after generating the task set, we randomly changed the properties of task type and made the HRCTs have the following ratio: 0, 33%, 50%, 66%, and 100%, corresponding to T0, T33, T50, T66 and T100 respectively. Under each utilization, we measured the energy consumption and the DMR of SRTs (deadline-missing task number of SRTs/total number of SRTs) at different HRCT ratio when using the HTDVS algorithm. The experiment results are shown in Figures 4 and 5 respectively.

Figure 4 shows that the five task sets consume more energy with increased utilization. With the increase in utilization, the TSFS-EN factors of tasks become smaller, and the dynamically reclaimed and reused energy also becomes small, which leads to high energy consumption. When the utilization is no more than 0.3, the energy consumption of T0-T100 is very close. Under those utilization, the energy savings of tasks mainly come from their static slowdown factors. Due to their bigger static slowdown factors, the G1-type subtasks and the G2-type subtasks except the final subtasks of HRCTs are all running at the lowest CPU speed. In these five task sets, the difference of energy consumption is mainly due to the difference of the energy consumption caused by the non-scaling final subtasks of HRCTs. When the utilization is greater than 0.3, the energy consumption of T0 is obviously lower than that of other task sets. The reason is all tasks in SRT can use their TSFS-EN factors and dynamically reclaim and reuse runtime slack without requiring meeting real-time requirements, which makes it use the energy-saving capability of tasks more effectively. Although we can also see T0 ≥ T66 ≥ T50 ≥ T33 ≥ T100 in energy consumption at most case, there are some abnormal points. For example, at the utilization of 0.7, T66 and T100 have similar energy consumption, and T33 has more energy consumption than that of T50. This is because the G1-type subtasks have the favorite running speeds and the reserved energy may not be fully used in the HTDVS algorithm. When the utilization is greater than 0.7, the energy consumption among T33-T100 is very close to each other. It means even little number of HRCTs will have strongly influence on the energy-saving effect in hybrid tasks systems with great utilization. It is because the hard real-time requirements of HRCTs should be guaranteed by restricting other tasks' slowdown factors.

Figure 5 shows the DMR of SRTs when performing the experiments in Figure 4. There is no T100 because there is no SRT in T100. Figure 5 shows the DMR of all four task sets is zero when the utilization is less than 0.4. It is because tasks have less WCET at lower utilization, the main energy savings come from their static TSFS-ENs. Because of the restriction of the lowest CPU running speed, their WCRT does not exceed their deadlines after using HTDVS. When the utilization is greater than 0.4, the DMR of T0 and T33 is bigger than zero. T0 has the largest DMR is because the tasks in T0 uses static TSFS-EN factors as their slowdown factors and dynamically reclaim and reuse slack time at runtime without mandatory requirements in meeting the deadlines of tasks, which will lead to much interference time among tasks. With the increment of utilization, the increment of interference time among tasks makes more tasks miss their deadlines. The DMR of T33 keeps stable from the utilization of 0.5. The increment of the DMR of T33 is mainly contributed by some SRTs which have less TSFS-EN factors and are easy to miss their deadlines, and these tasks do not increase with the increment of utilization (of course, whether the DMR of tasks will increase is dependent on the distribution of TSFS-EN factors of tasks.). The DMR of T50 and T66 is always zero in Figure 5 because more HRCTs in the two task sets exert more restriction on the slowdown factors of tasks and their dynamic slack time reuse. Note that although the values of the DMR in Figure 5 have something to do with the subtasks' structure and TSFS-EN distribution, more HRCTs will lead to lower DMR.

### 6.2. Different Energy Reuse Algorithms

**Figure 6.**
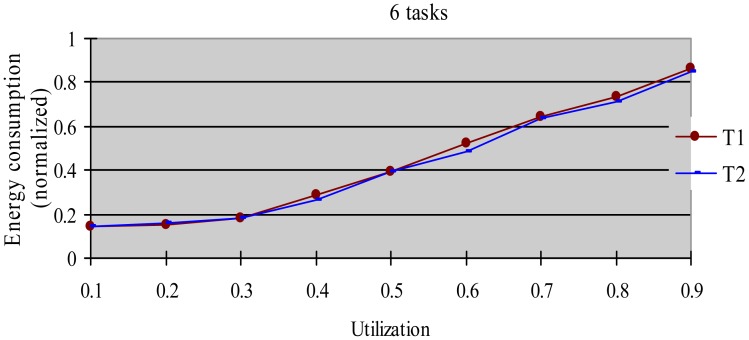
Normalized unweighted energy consumption when using different energy-saving algorithms.

**Figure 7.**
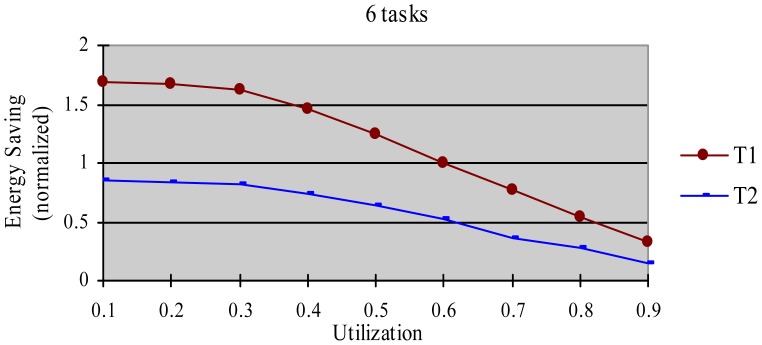
Normalized weighted energy consumption when using different energy-saving algorithms.

The greedy algorithm is a widely used dynamic energy reclamation and reuse algorithm [8,20,36], and has proved its good energy-saving effects. In this experiment, we compared the energy-saving effect of the HTDVS algorithm to that of the greedy algorithm. We use a randomly generated task set with 50% HRCTs and 50% SRTs. Because the subtasks in HoW have different function weight, we compare their energy-saving effect in two cases, *i.e.*, without weight and with weight respectively. When not considering weight, we believe the energy-saving benefit is the same among all subtasks, and compared energy consumption ratio between the HTDVS algorithm and the greedy algorithm. When considering weight, we compared the weighted energy consumption between the HTDVS algorithm and the greedy algorithm. The weighted energy consumption of a task is the sum of the multiplication of energy-saving energy of each subtask and the function weight of each subtask. In Figure 6, T1 denotes the energy consumption when the HTDVS algorithm is used, and T2 denotes the energy consumption when the greedy algorithm is used. The experiment results are shown in Figures 6 and 7, respectively.

Figure 6 shows the energy consumption increase with the increment of utilization whether using the HTDVS algorithm or the greedy algorithm. The lowest energy consumption in T1 and T2 is about 18% due to the same reason as in experiment 1. When the utilization is greater than 0.3, the energy consumption of T1 is usually more than that of T2 because the reserved energy in the HTDVS algorithm may not be fully used, and the G1-type subtasks can only use some specific energy, while the subtasks can try its best to use any slack time in the greedy algorithm. However, the energy-saving effect is very close between T1 and T2 at the most case, which proves the HTDVS algorithm have good energy-saving effect.

Figure 7 shows the energy-saving effect of T2 is better than that of T1 when we consider the function weight. It is because the energy-reserving mechanism in the HTDVS algorithm can serve for subtasks with larger function weight, and obtain more energy-saving benefit.

### 6.3. Different Subtask Type Ratio

**Figure 8.**
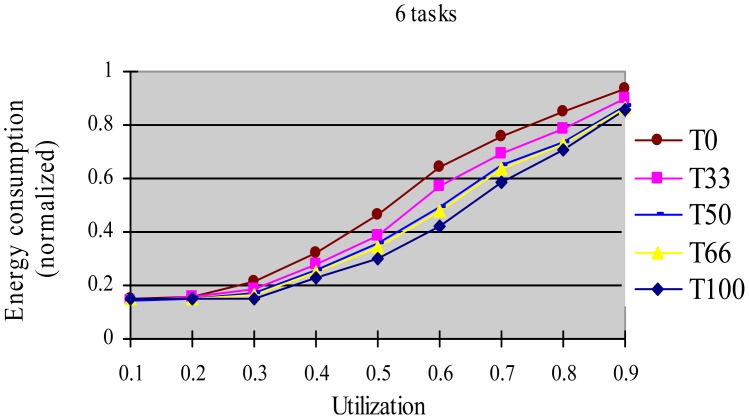
Normalized energy consumption at different subtask type ratio.

In this experiment, we use a randomly generated task set with 50% HRCTs and 50% SRTs. After generated the task set, we randomly change the energy-saving goals of subtasks and make the tasks with G2-type energy-saving goals have the following ratio: 0, 33%, 50%, 66%, and 100%, corresponding to T0-100 respectively. Under each utilization, we measure the energy consumption when using the HTDVS algorithm. The experimental results are shown in Figure 8.

Figure 8 shows the energy consumption increase with the increment of utilization. When the utilization is less than 0.2, the energy consumption of all five task sets is restricted by the lowest CPU energy consumption and the energy consumption of the final subtasks of HRCTs. Because the lowest CPU energy consumption is the same and the energy consumption of the final subtasks of HRCTs is very close, the energy consumption of the five task sets is almost equal. When the utilization is greater than 0.3, the energy consumption of T0, T33, T50, T66, and T100 is in descending order. It is because the G2-type subtasks can use dynamic slack time effectively compare to the G1-type subtasks with specific favorite running speeds. More G2-type subtasks can fully use dynamic slack time to lower the subtasks' speeds, and obtain better energy-saving effect.

## 7. Conclusions and Future Work

Aiming at the energy-saving problem for IoT control devices, we present the HoW task model and an energy-saving method called HTDVS. Experimental results show the HTDVS algorithm is influenced by the ratio of HRCTs and the energy-saving goals of subtasks, and it has similar energy-saving effect with the greedy algorithm. Our future work will focus on: (1) how to extend HoW to model aperiodic tasks and sporadic tasks, extend the processor model to multiple core processors, and integrate the new system model into HTDVS; (2) how to accelerate specific SRTs to meet their DMR under a given threshold; and (3) how to integrate other constraints, such as temperature, into energy-saving algorithm in order to avoid errors when software runs in extreme environment.
